# Integrating Agronomic Traits and Physiological Responses for Drought Resistance Screening in Wheat Germplasms

**DOI:** 10.3390/plants15040576

**Published:** 2026-02-12

**Authors:** Wenwen Cui, Yan Jin, Baoyuan Zhou, Liang Chen, Jiajing Song, Quanhao Song

**Affiliations:** 1 Zhumadian Academy of Industry Innovation and Development, Huanghuai University, Zhumadian 463000, China; 2 Zhumadian Academy of Agricultural Sciences, Zhumadian 463000, China; 3 Institute of Crop Sciences, Chinese Academy of Agricultural Sciences, Beijing 100081, China; 4 State Key Laboratory for Crop Stress Resitance and High-Efficiency Production, College of Agronomy, Northwest A&F University, Yangling 712100, China

**Keywords:** drought resistance, chlorophyll fluorescence, antioxidant enzyme, wheat germplasm, membership function value

## Abstract

Drought stress is a critical limiting factor for wheat yield. Wild relatives of wheat have proven to be valuable genetic resources for desirable traits. This study aimed to conduct a comparative analysis of agronomic traits, photosynthetic physiological parameters, and antioxidant components among 26 heterogermplasm wheat cultivars under well-watered (WW) and water-stressed (WS) conditions over two consecutive years. The results revealed that all nine agronomic traits were adversely affected under WS conditions. Four agronomic traits were selected based on the drought-resistance coefficient (DC < 0.8) and heritability (*H*^2^ < 0.7) to calculate the membership function value of drought resistance (MFVD), including flag leaf area (FLA), tiller number (TN), grain yield per plant (GYPP), and biomass per plant (BMPP). All wheat genotypes clustered into three groups based on their mean value of MFVD in two years. Under drought stress, wheat germplasms classified within the high MFVD group demonstrate significantly enhanced drought adaptability, as evidenced by superior photosynthetic performance with elevated photosynthesis rate (Pn), the actual photochemical quantum efficiency of photosystem II (ΦPSII), and the electron transfer rate (ETR), increased chlorophyll retention (higher SPAD values), strengthened antioxidant enzyme activities, and reduced stomatal limitation. Correlation analyses further reveal that MFVD exhibits significant positive correlations with Pn, ΦPSII, ETR, SPAD, and key antioxidant enzymes, while displaying a significant negative correlation with stomatal limitation value (Ls). These consistent physiological and biochemical patterns corroborate that the constituent agronomic traits—tiller number (TN), flag leaf area (FLA), biomass per plant (BMPP), and grain yield per plant (GYPP)—serve as robust and integrated phenotypic indicators for comprehensively evaluating drought resistance in wheat germplasm. Among the evaluated lines, lines 6, 15, 17, 21, and 22 exhibited significantly higher levels of drought resistance. These results highlight the presence of genetic variability among heterogermplasm wheat cultivars, which can be harnessed in breeding programs to develop drought-tolerant wheat varieties.

## 1. Introduction

Wheat (*Triticum aestivum* L.) is one of the most important cereal crops globally, with a wide latitudinal distribution across the world. Climate change is expected to increase the occurrence of extended dry periods [1]. Drought stress has been reported to induce a wide range of biochemical and physiological modifications in plants [2,3]. As water deficit increasingly constrains wheat production worldwide, there is an urgent need to identify drought-tolerant donor genotypes for integration into locally adapted landraces.

Morphological and physiological responses of crops to drought stress can account for the substantial yield variability observed under water-deficit conditions [4]. From a morphological standpoint, drought stress induces a reduction in the size of key wheat organs—including leaves, stems, ears, and tillers, which consequently leads to decreases in plant height, panicle number, spikelet fertility rate, thousand-grain weight (TGW), and aboveground biomass accumulation [5,6]. These alterations cause the abortion of reproductive structures, as well as a decrease in the accumulation of biomass in storage organs, causing losses in crop production [7,8]. Previous studies have demonstrated that plant biomass, dry matter accumulation, plant height, tiller number, spikelet number, grains per spikelet, and thousand-grain weight are sensitive to water stress. Furthermore, biomass-related traits are significantly correlated with grain yield under water-limited conditions and have thus been widely adopted as key evaluation parameters for drought resistance [9,10,11].

In addition to the phenotypic alterations induced by drought stress, crops undergo extensive physiological and biochemical adjustments to mitigate the adverse effects of environmental stress. Photosynthesis, as the fundamental process underlying plant growth and the primary metabolic pathway in plants, is susceptible to disruption even under mild stress conditions [12]. Under water-stress conditions, the typical response of plants is stomatal closure, reducing equivalents generated by photosynthesis are in excess in chloroplasts, which could not only cause structural damage to PSII and light harvesting complexes directly, but also affect the process of photosynthetic electron transport, photophosphorylation, and accumulation of reactive oxygen species (ROS), resulting in photoinhibition [13,14,15]. Meanwhile, chlorophyll content is a key factor in maintaining the stability of the photosynthetic system. Studies have demonstrated that elevated chlorophyll content during the grain-filling stage constitutes a reliable indicator for screening winter wheat cultivars adapted to dryland environments [16]. Various biophysical parameters derived from Chla fluorescence transient measurements are a potential and simple tool to evaluate the impact of drought stress on the photosynthetic apparatus, help to understand the energy flow through PSII, and provide useful indicators of the development and severity of stress effects [17,18]. The maximum quantum yield of primary photochemistry (Fv/Fm) in leaves in the dark-adapted state can reflect wheat’s light-use efficiency under drought conditions [19]. Reactive oxygen species (ROS) are implicated as a critical mechanism contributing to the enhanced adaptation of plants to adverse environmental conditions [20]. The production of ROS can cause chemical damage to DNA and proteins, interfering with a series of physiological and biochemical processes [21,22,23]. Thus, physiological and biochemical parameters—including photosynthetic rate, chlorophyll content, and antioxidant enzyme activities (SOD, POD, and CAT)—represent reliable indicators for the evaluation of drought resistance [24,25].

Currently, research on drought resistance evaluation in wheat primarily focuses on germination-related indices [26], seedling growth traits [27,28], agronomic traits [29], high-throughput spectral indices [30,31], yield-related characteristics [32], as well as molecular marker techniques such as ISSR and SCoT PCR combined with agronomic traits [33]. However, studies integrating photosynthetic fluorescence parameters and reactive oxygen species metabolism during the reproductive growth stage with mature-stage agronomic traits for drought resistance analysis remain relatively scarce. The reproductive growth stage is critical for wheat yield formation. During this phase, photosynthetic fluorescence characteristics and reactive oxygen species metabolism directly reflect the physiological response mechanisms of wheat under drought stress, which holds significant importance for a deeper understanding of wheat drought resistance. Therefore, the objective of this study was to evaluate the drought resistance of multiple wheat germplasm resources by integrating agronomic traits at the mature stage with physiological indicators measured during the reproductive growth phase, including drought-sensitive photosynthetic fluorescence parameters and antioxidant enzyme activities, in order to identify promising germplasm for future breeding applications.

## 2. Results

### 2.1. Descriptive Statistics of Agronomic Traits Under Drought Conditions

Nine agronomic traits of 26 wheat germplasms were measured at maturity (Table 1), and descriptive statistical analyses were performed for each trait. The results demonstrated that in the WS condition, the coefficient of variation (CV) for measured traits ranged from 13.4% to 40.3%, whereas under WW conditions, the CV of the investigated traits ranged from 13.3% to 34.4%. Among these, the CV for all indices exceeded 20%, with the exception of PH, UIL, TGW, and GNPS. In WS conditions, the maximum, minimum, and mean values of all measured parameters were lower than those under WW conditions, demonstrating a systemic physiological recession under drought stress.

### 2.2. Genetic Variation and Broad Sense Heritability (H^2^) of Traits

Analysis of variance (ANOVA) for all nine agronomic traits examined revealed highly significant differences among both gene and water treatments (Table 2). The nine traits exhibited significant variations in both years, with the exception of PH. Environmental factors such as soil moisture, temperature, and their interaction may attribute to this phenomenon. The *H*^2^ of all traits exhibited a wide range, varying from 0.53 to 0.93, with the highest *H*^2^ value observed for PH at 0.93, followed by UIL and GNPS at 0.89 each. A higher heritability (*H*^2^ > 0.7) indicates that the observed variation in these characteristics among the tested wheat materials was primarily attributed to genetic factors and remained highly stable across all environments. The *H*^2^ values of FLA, TN, GYPP, and BMPP were less than 0.7, which were 0.53, 0.54, 0.62, and 0.56, respectively. Low heritability suggests that these traits among the tested materials are highly susceptible to environmental influences.

### 2.3. Evaluation of Drought Sensitivity in Traits Based on the DC

During two consecutive growing seasons, water stress induced a significant reduction in the mean values of all nine agronomic traits relative to the WW control, such that the DC was consistently less than 1 across all traits (Table 3). GYPP was the most sensitive trait to water stress, with DC of 0.58 and 0.70 across the two growing seasons, indicating the most severe drought-induced impairment and thus the weakest drought resistance among all investigated traits. This was followed by BMPP, with DC values of 0.64 and 0.76 in the two seasons. The DC of FLA and TN also fell below 0.8, whereas that of the remaining traits exceeded this threshold. TGW exhibited the strongest drought resistance, with DC values greater than 0.95 over the two-year experimental period, demonstrating enhanced drought tolerance and superior phenotypic stability under water-stressed conditions in these wheat germplasms.

### 2.4. Evaluation of Drought Resistance by MFVD

**Figure 1:**
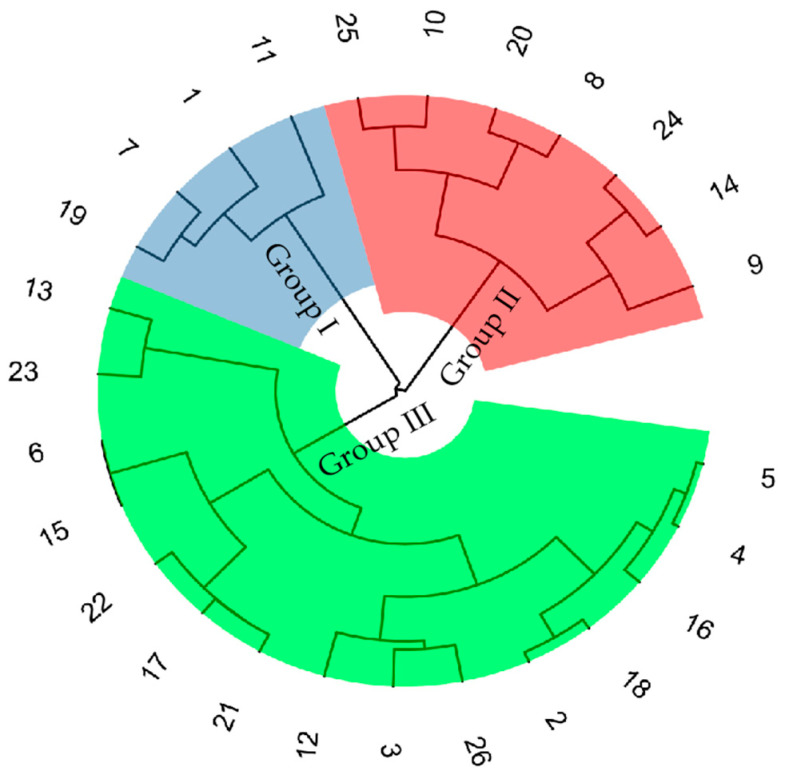
Cluster map of 26 wheat varieties.

**Figure 2:**
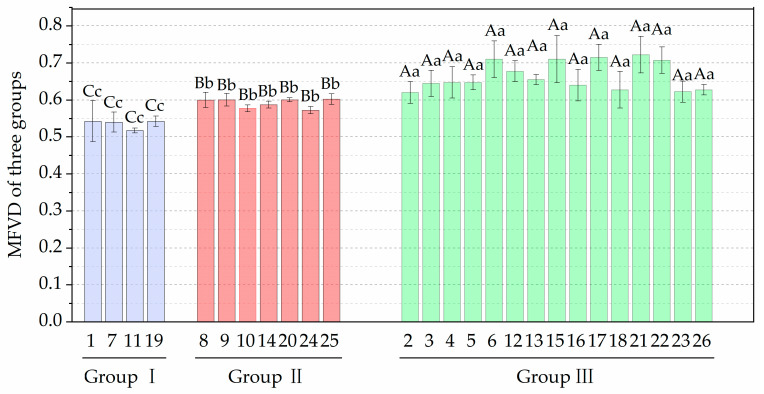
Membership function value of drought resistance (MFVD) of the three groups of experimental materials. Vertical bars indicate ± standard error of the mean. Significant differences among different water regimes are indicated by different lowercase (*p* < 0.05) and uppercase (*p* < 0.01) letters, Duncan, respectively.

Five agronomic traits (PH, UIL, DSL, GNPS, and TGW) were excluded from subsequent analyses, based on their consistently high drought resistance coefficients (DC > 0.8) and higher heritability (*H*^2^ > 0.7) under WS conditions, indicating minimal drought-induced phenotypic alterations. Four agronomic traits (FLA, TN, GYPP, and BMPP) with lower DC (DC < 0.8) and heritability (*H*^2^ < 0.7) were used to calculate the MFVD for each line. Hierarchical clustering analysis was subsequently performed using the MFVD values derived from two consecutive growing seasons, employing Ward’s method with Euclidean distance metrics. All wheat genotypes were clustered into three distinct groups based on the mean values of MFVD across the two experimental years (Figure 1). Based on the clustering results of the mean MFVD, multiple comparisons were conducted among high, moderate, and low-drought resistance groups (Figure 2). Four wheat germplasms in Group I exhibited relatively low MFVD levels (0.51–0.54), while Group II, with seven wheat germplasms, displayed moderate MFVD values (0.57–0.60). Highly significant differences (*p* < 0.01) were observed between these two groups. Fifteen wheat germplasms (2, 3, 4, 5, 6, 12, 13, 15, 16, 17, 18, 21, 22, 23, and 26) in group III had significantly higher MFVD (0.62–0.72) than the other two groups, suggesting they have good drought resistance. The MFVD values of five wheat germplasm (6, 15, 17, 21, and 22) in group III exceeded 0.7, indicating that they have stable higher drought resistance (Figure 2).

### 2.5. Physiological and Biochemical Analysis of Three MFVD Groups of Wheat Germplasms

#### 2.5.1. Antioxidant Enzyme Activities

**Figure 3:**
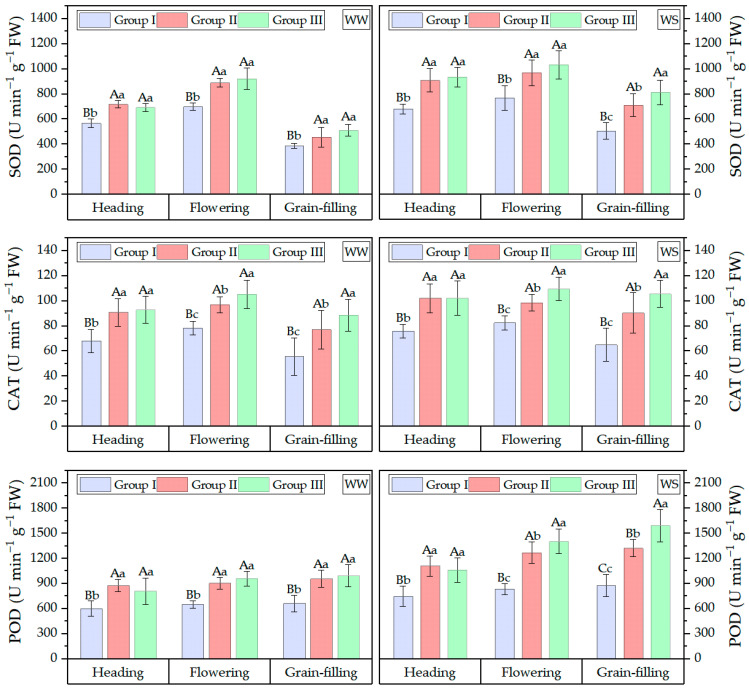
Activities of antioxidant enzymes in flag leaves of three MFVD-clustered drought resistance groups at three primary growth stages under WW and WS treatment. Different lowercase and uppercase letters represent difference significant among the different water conditions at (*p* < 0.05) and (*p* < 0.01), Duncan.

To further elucidate the contribution of antioxidant enzyme activities in flag leaves to drought resistance, the activities of SOD, POD, and CAT were determined at three key growth stages and compared across the three MFVD-based groups (Figure 3). With the development of the growth process, the activity of CAT and SOD in the flag leaf were exhibited a trend of first increasing and then decreasing across the three growth stages in all three groups, while the activity of POD exhibited an increasing trend. In WS treatments, the enzyme activities in the flag leaf of all genotypes were significantly higher than those under WW conditions. The material of Group I with the lowest MFVD value exhibited the lowest activities of CAT, SOD, and POD enzymes at all growth stages. During the flowering and grain-filling stages, Group II materials demonstrated moderate enzyme activities, whereas Group III materials showed significantly higher enzyme activities compared to other groups. At the heading stage, the enzyme activities of Group II materials were slightly higher than those of Group III, although the difference was not statistically significant. Wheat varieties in Group III with higher antioxidant enzyme activity typically exhibit enhanced stress resistance. The elevated antioxidant enzyme activities in Group III materials indicate their superior stress resistance, enabling them to maintain normal growth and development under stress conditions.

#### 2.5.2. Photosynthesis-Related Parameters

The role of photosynthetic physiological characteristics in flag leaves was analyzed to elucidate their contribution to the drought resistance of the wheat germplasm (Table 4). Under WW conditions, no significant differences were observed among the three different MFVD groups for three physiological characters (Pn, Ls, and WUE) at contemporaneity. However, the Group III with high drought-resistance showed a higher photosynthetic rate, especially at the grain-filling stage; the photosynthetic rate of Group III was 14.4% and 15.3% higher than that of Group I and Group II, respectively. Under WS conditions, Group III exhibited a significantly higher photosynthetic rate compared to the other two groups at the same developmental stage. The average photosynthetic rate of Group III was 36.2% and 19.35% higher than that of Group I and Group II, respectively. However, the Ls demonstrated a U-shaped dynamic pattern during three stages, characterized by higher initial values at the heading stage, a significant decline to a minimum at the flowering stage, and subsequent recovery during grain-filling. Notably, Group III exhibited attenuated fluctuations in Ls across these stages, showing a stable stomatal conductance. The chlorophyll content also varied among the different drought resistance MFVD value groups. Regardless of WS or WW conditions, all three groups exhibited a consistent chlorophyll content gradient that aligned with their drought resistance hierarchy: Group III, with high drought resistance, displayed the highest SPAD values, followed by Group II (moderate resistance), while Group I (low resistance) showed the lowest levels.

#### 2.5.3. Chlorophyll Fluorescence-Related Parameters

Whether under WW or WS conditions, no significant differences were observed in the six simultaneously measured fluorescent parameters among the three MFVD groups at the same period (Table 5). The highest value of Fv′/Fm′ was observed in Group I, but there was no significant difference among all three groups. Under WS conditions, significantly lower and higher ΦPSII existed in Group I and Group III at all three stages. Analysis of the ETR, Group I displayed the lowest ETR, and Group III displayed the highest ETR at all three stages. Group III with high drought resistance displayed significantly higher qP and ETR at both heading and grain-filling stages, while group I exhibited the lowest qP and ETR value. There was no significant difference revealed among various drought resistance for qN and Fv/Fm, but the maximum value was expressed in the high MFVD group III for qN.

### 2.6. Correlations Between MFVD and Physiological and Biochemical Indicators

#### 2.6.1. Correlations Between MFVD with Photosynthetic and Fluorescence Parameters

Correlation coefficients between MFVD and Pn, Ls, and fluorescence parameters varied across different growth stages are shown in Table 6. Under WW conditions, no significant correlations were observed between MFVD and the measured indices. In contrast, under WS conditions, MFVD showed significant positive correlations with Pn across all three growth stages, with correlation coefficients (r) ranging from 0.631 to 0.653. The negative correlation between MFVD and Ls (r = −0.292 to −0.351) indicates that materials with high MFVD possess superior stomatal regulation capabilities. Furthermore, the positive correlations of MFVD with both ΦPSII and ETR further indicate that materials with higher MFVD values can maintain more effective photosystem operation under stress.

#### 2.6.2. Correlations Between MFVD with Antioxidant Enzyme Activity and SPAD

The correlation patterns between MFVD and antioxidant enzyme activities differed among growth stages (Table 7). Under WW conditions, SOD activity exhibited a significant positive correlation with MFVD exclusively at the grain-filling stage (r = 0.399). In contrast, CAT and POD activities were significantly and positively correlated with MFVD at the heading, flowering, and grain-filling stages. Under WS conditions, significant positive correlations were consistently observed between MFVD and the activities of CAT, POD, and SOD across all three growth stages, with correlation coefficients (r) ranging from 0.529 to 0.694. The SPAD value was significantly correlated with MFVD under WS conditions at the heading (r = 0.327), flowering (r = 0.351), and grain-filling (r = 0.388) stages. However, no significant correlation between SPAD values and MFVD was detected under WW conditions throughout the wheat growth cycle. These results demonstrate that genotypes with high MFVD can synergistically enhance the antioxidant defense system and maintain photosynthetic pigment stability under drought stress, indicating that antioxidant enzyme activities and chlorophyll content play crucial roles in the drought resistance mechanisms of wheat germplasm.

## 3. Discussion

The selection of traits was based on clear physiological and genetic principles. FLA, TN, GYPP, and BMPP all exhibited relatively low drought coefficients (DC < 0.8) under drought stress, indicating their high sensitivity to water deficit and their effectiveness in reflecting genotypic differences in drought resistance. In contrast, traits such as PH and UIL, which showed high DC values (DC > 0.8) and high heritability, remained stable under drought and are thus more suitable as background reference traits. This strategy of trait selection based on “stress-response sensitivity” aligns with the mainstream approach in crop drought evaluation, which prioritizes plastic traits over constitutive ones [34,35]. Furthermore, FLA, as a key indicator of the size of the photosynthetic source organ, is crucial for light capture and carbon assimilation under drought conditions [36]. TN and BMPP jointly characterize the plant’s capacity for population establishment and biomass allocation under stress, with their dynamic changes representing an important adaptive strategy to drought [37,38]. GYPP, as the ultimate reproductive output, serves as the primary agronomic target for evaluating drought resistance [39,40]. Therefore, this index system logically encompasses the key stages of drought response, from morphological plasticity and growth development to yield formation.

The high-MFVD genotypes demonstrated enhanced physiological performance under water deficit conditions, characterized by significantly higher Pn, ΦPSII, and ETR, alongside elevated activities of key antioxidant enzymes—SOD, POD, and CAT—as well as greater chlorophyll content (indicated by SPAD values). A notable negative correlation was observed between these genotypes and Ls, reflecting more efficient stomatal regulation during drought stress. These results align with previous reports indicating that drought-tolerant wheat genotypes generally retain higher pigment content, exhibit better photosynthetic performance, improved chlorophyll fluorescence parameters, optimized stomatal regulation, and stronger antioxidant enzyme activities compared to drought-sensitive lines [41,42,43].

Consistent with prior findings, drought-resistant germplasm exhibits a prompt and precise activation of the antioxidant defense system, enabling tight regulation of reactive oxygen species (ROS) levels [44]. This regulatory capacity confers a dual physiological advantage: first, the rapid scavenging of excess ROS, mitigates oxidative inhibition of key enzymes in photosynthetic carbon assimilation, such as those involved in the Calvin cycle [45]; second, moderately retained H_2_O_2_ functions as a signaling molecule that suppresses abscisic acid (ABA) accumulation in leaves, thereby mitigating stomatal limitation, reducing Ls, and improving WUE [46,47]. Although drought stress typically induces chlorophyll degradation [48], the high-MFVD group maintained higher SPAD values, indicating a superior ability to preserve photosynthetic pigments. Concurrently, chloroplastic SOD played a crucial role in protecting photosystem II from oxidative damage by detoxifying superoxide anions, which helped sustain Fv/Fm and ΦPSII [49]. The observed increases in ETR and qP further suggest that these genotypes utilize captured light energy more efficiently for photochemistry, thereby supporting carbon assimilation. Moreover, the maintenance of moderate qN in the high-MFVD group points to an effective dissipation of excess excitation energy, alleviating photoinhibition and stabilizing Pn under stress. POD directly reduces lipid peroxides and acts synergistically with the SOD–CAT pathway to significantly lower the accumulation of malondialdehyde (MDA), a marker of membrane lipid peroxidation [50]. This coordinated action helps maintain membrane integrity and cellular function under drought conditions. Collectively, the high-MFVD wheat germplasm appears to employ a coordinated drought adaptation strategy involving (1) synergistic upregulation of antioxidant defense, (2) optimized stomatal behavior, (3) maintenance of photosynthetic machinery function and stability, and (4) protection of membrane integrity. This integrated physiological response enables a more favorable balance between growth and productivity under water-limited conditions.

The systematic positive correlations of MFVD with photosynthetic parameters (Pn, ΦPSII, ETR), chlorophyll content (SPAD), and antioxidant enzyme activities, coupled with its negative correlation with Ls, delineate an integrated drought resistance pathway spanning from morphological maintenance (FLA, BMPP), through physiological function (photosynthetic performance and antioxidant defense), to final agronomic output (GYPP). Mechanistically, stable flag leaf area supports the structural and functional integrity of the photosynthetic apparatus, thereby mitigating photoinhibition under stress [36]; the dynamic regulation of biomass accumulation and tillering (TN) reflects strategic carbon partitioning and resource allocation that balances survival and reproductive investment under combined light and water limitations [37,38]; and the coordinated upregulation of antioxidant enzymes constitutes a fundamental biochemical response to drought-induced oxidative stress [38]. Therefore, the MFVD index, synthesized from FLA, TN, GYPP, and BMPP, provides not only an agronomically tractable and comparable phenotypic metric but also a physiologically integrated proxy that captures key drought-adaptive dimensions—including source–sink coordination, water-use efficiency, oxidative stress tolerance, and yield stability—at a functional level.

## 4. Materials and Methods

### 4.1. Plant Materials

Twenty-six wheat germplasm accessions were selected for this study (Table 8), representing five distinct genetic backgrounds: four bread wheat cultivars (BC), four synthetic hexaploid wheat lines (SHW), ten disomic alien chromosome addition lines (CA) derived from Chinese Spring, four hexaploid triticale lines (HT), and four octoploid triticale lines (OT). All materials were chosen based on comparable phenological traits from a preliminary screening of 108 germplasm accessions.

### 4.2. Field Experiments and Water Treatments

The field experiments were carried out over two consecutive winter growing seasons (October to June in 2022–2023 and 2023–2024) in the rain-out shelter of the Institute of Water Saving Agriculture in Arid Regions of China, Northwest A&F University, Yangling (34°20′ N, 108°04′ E, 454.8 m altitude), Shaanxi, China. The rain-out shelter remained open unless precipitation occurred. Materials were planted in a randomized complete block design with three replications; each genotype was planted in three rows with 1.8 m length, 25 cm between rows, and 6.67 cm between plants under both well-watered (WW) and water-stressed (WS) conditions. The experimental soil was classified as sandy loam, with the following chemical properties (per kg dry soil): organic matter, 10.75 g; total N, 1.51 g; total P, 0.80 g; total K, 5.83 g; available N, 57.90 mg; available P, 26.21 mg; and available K, 96.75 mg. Throughout the growth period, diseases, pests, weeds, and fertilizer in all treatments were managed according to standard agricultural practices to minimize their impact on crop growth.

Both WW and WS plots were fully irrigated prior to sowing to ensure uniform seedling establishment. Irrigation was applied using a sprinkler system throughout the growing season. Across the two experimental years, a total irrigation amount of 220 mm was applied to the well-watered (WW) plots, while 145 mm was supplied to the water-stressed (WS) plots. During the tillering and stem elongation stages, 50 mm of irrigation was applied to both the WW and WS plots. Subsequently, at the heading, flowering, and grain-filling stages, the WW plots received 40 mm of irrigation per stage, whereas the WS plots received 15 mm per stage. At physiological maturity, the average gravimetric soil moisture content (0–100 cm depth) in the 2022–2023 and 2023–2024 growing seasons was 12.87% and 12.49% under WW conditions and 10.63% and 10.21% under WS conditions, respectively.

### 4.3. Agronomic Traits Investigation

At maturity, ten plants were randomly sampled from each replicate per material. The following yield-related agronomic traits were recorded: plant height (PH), length of the first internodes under the spike (UIL), distance from spike to flag leaf ligule (DSL), flag leaf area (FLA), tiller number (TN), and grain number spike^−1^ (GNPS). After sun-drying, biomass plant^−1^ (BMPP), grain yield plant^−1^ (GYPP), and thousand kernel weight (TGW) were measured.

### 4.4. Assays for Antioxidant Enzyme Activity

The activities of superoxide dismutase (SOD), peroxidase (POD), and catalase (CAT) were assayed at heading, flowering, and grain-filling stages. Then, 0.4 g fresh leaves were weighed and ground with liquid nitrogen in 4 mL of precooled sodium phosphate buffer (0.05 mol/L Na_2_HPO_4_-NaH_2_PO_4_, pH 7.8) and then centrifuged at 12,000× *g* for 20 min. The supernatant was used as the enzyme extract for measuring enzyme activity using the TECAN GENIOS (Tecan Infinite 200 PRO, Tecan Corp., Männedorf, Switzerland). SOD activity was determined by recording the decrease in the absorbance of nitro-blue tetrazolium (NBT) by the enzyme [51]. POD activity was assayed using the guaiacol method according to Ekmekci and Terzioglu [52]. CAT activity was assayed according to Zhao et al. [53].

### 4.5. Assessment of Photosynthesis Characteristics and Chlorophyll Fluorescence

#### 4.5.1. Chlorophyll

Chlorophyll content was estimated using a portable chlorophyll meter (SPAD-502, Minolta Camera Co., Osaka, Japan). Measurements were taken on the uppermost fully expanded leaves, and thirty SPAD readings per plot were averaged to obtain a mean plot value. Healthy flag leaves were selected for determination at heading, flowering, and grain-filling stages.

#### 4.5.2. Photosynthetic and Chlorophyll Fluorescence Parameter

Photosynthetic parameters including net photosynthesis rate (Pn), internal CO_2_ concentration (Ci), stomatal conductance (gs), transpiration rate (Tr), and chlorophyll fluorescence parameters, including fluorescence maximum (Fm), fluorescence origin (F0), variable fluorescence (Fv), photochemical quenching coefficient (qP), non-photochemical quenching coefficient (QN) were determined with a portable Li-6400XT photosynthesis system (Li-Cor; Lincoln, NE, USA), requiring a fluorescence probe to measure the chlorophyll fluorescence parameters. Measurements were carried out from 9:00–11:00 a.m. on a sunny day under ambient conditions (irradiation up to 1200 μmol m^−2^ s^−1^, 400 μmol mol^−1^ CO_2_ concentration, 20 °C). After the leaves were fully dark-adapted, the chlorophyll fluorescence parameters were measured on the same leaf. The measurements were conducted by selecting ten healthy flag leaves in each treatment at heading, flowering, and grain-filling stages.

### 4.6. Data Analysis

All agronomic traits under both WS and WW conditions were subjected to analysis of variance (ANOVA) using SAS 8.0 (SAS Institute, Cary, NC, USA). Data organization and preliminary processing were performed in Microsoft Excel 2019 (Microsoft; Redmond, WA, USA). Cluster analysis was carried out in RStudio (R version 4.3.1) with the factoextra package (v1.0.7). The broad sense heritability (*H*^2^) of each trait was calculated as follows:

(1) $$H^{2}=\frac{Vg}{V_{g}\;+\;V_{gw}\;+\;V_{gy}-V_{gwy}+V_{\varepsilon }}$$

where V_g_ represents the genotypic variance; V_gw_ denotes the genotype × water treatment interaction variance; V_gy_ indicates the genotype × year interaction variance; V_gwy_ refers to the genotype × water treatment × year interaction variance; and V_ε_ is the error variance. The drought-tolerance coefficient (DC) [54,55], membership function value of drought resistance (MFVD) [56], and drought resistance index (DRI) [57] were calculated as follows:

(2) $${DC}_{i\cdot j}=\frac{X_{i\cdot j\cdot ws}}{X_{i\cdot j\cdot ww}}$$

(3) $$U_{i\cdot j}=\frac{{DC}_{i\cdot j}-{DC}_{jmin}}{{DC}_{jmax}-{DC}_{jmin}}$$

(4) $${MFVD}_{i}=\frac{1}{n}\sum_{j=1}^{n}U_{i\cdot j}$$

where DC_i·j_ represents the drought-tolerance coefficient of genotype (i) for trait (j); X_i·j·ws_ and X_i·j·ww_ denote the phenotypic values of trait (j) for genotype (i) under WS and WW conditions; U_i·j_ indicates the membership function value of drought resistance for genotype (i) regarding trait (j); DC_jmax_ and DC_jmin_ are the maximum and minimum DC values observed across all genotypes for trait (j); MFVD refers to the arithmetic mean of the membership function values of drought resistance across all target traits.

## 5. Conclusions

Four agronomic traits (FLA, TN, GYPP, and BMPP) can serve as effective indicators for the evaluation of drought resistance in wheat. The materials in the Group III showed better reactive oxygen species regulation and photosynthetic functions under drought conditions, especially the five materials (6 Synthetic hexaploid wheat, 15 Chinese Spring-Aegilops caudata Disomic ?C addition, 17 Chinese Spring-Rye Disomic addition 1R, 21 Hexaploid Triticale, and 22 Hexaploid Triticale) with high MFVD values (>0.7), indicated them with a stable higher drought resistance. These results demonstrate the potential of hetreogermplasm wheat as a source for improving drought resistance. Future studies should integrate genetic analyses with multi-environment drought-phenotyping to systematically elucidate the genetic basis of drought resistance in these materials.

## Figures and Tables

**Table 1:** Descriptive statistics of nine agronomic traits of 26 wheat germplasm resources.

Year	WT	Item	PH (cm)	UIL (cm)	DSL (cm)	FLA (cm^3^)	TN	GNPS	TGW (g)	GYPP (g)	BMPP (g)
2022–2023	WW	Min	68.9	18.3	5.5	9.9	3.3	28	27.6	5.5	16.3
		Max	160.5	54.1	32.6	40.9	7.7	64.7	50.1	34.1	74.1
		Mean	123.7	39.6	20.3	22.9	5.5	48.3	39.9	12.5	33.4
		SD	17.9	6.6	4.2	6.6	1.2	7.9	5.3	4.3	6.8
		CV (%)	14.5	16.7	20.7	28.8	21.8	16.4	13.3	34.4	20.4
	WS	Min	66.1	16.4	3.8	7.9	2.5	21.7	26.6	2.6	10.2
		Max	145.4	50.3	30.6	32.1	6.3	57.7	48.5	15.3	32.8
		Mean	115.2	35.4	17.3	17.1	4.3	41.6	38.2	7.2	21
		SD	17.5	6.2	3.7	5.7	1.1	6.9	5.1	2.9	5.1
		CV (%)	15.2	17.5	21.4	33.3	25.6	16.6	13.4	40.3	24.3
2023–2024	WW	Min	63	19.8	2.8	30.3	5.8	36.7	27.1	6.7	23
		Max	156.6	52.9	31.7	70.5	21	85.7	48.4	23.3	94.2
		Mean	124.8	40.6	19	45.3	13.1	60.3	35.1	14.5	47.9
		SD	20	7.8	4.3	8.9	3.1	10.3	4.8	4	11.9
		CV (%)	16.0	19.2	22.6	19.6	23.7	17.1	13.7	27.6	24.8
	WS	Min	57.8	16.7	2.2	23.3	3.9	32.1	23.3	4.3	15.3
		Max	146.4	51.3	29.6	60.1	16.3	76.7	46.6	20.5	64.3
		Mean	115.2	36.9	15.9	33.1	10	53.9	33.2	10.1	36.6
		SD	20.2	7.1	3.8	7.7	3	9.7	5.1	3.6	11
		CV (%)	17.5	19.2	23.9	23.3	30.0	18.0	15.4	35.6	30.1

WT, water treatment; WW, well-watered; WS, water-stressed; Min, minimum; Max, maximum; CV, coefficient of variation; PH, plant height; UIL, length of the first internodes under the spike; DSL, distance from spike to flag leaf ligule; FLA, area of flag; TN, tiller number; GNPS, number of grains spike^−1^; TGW, thousand-grain weight; GYPP, grain yield plant^−1^; BMPP, biomass yield plant^−1^.

**Table 2:** Analysis of ANOVA and broad-sense heritability (*H*^2^) for the agronomic traits investigated across the two-year experimental period.

Source	DF	PH	UIL	DSL	FLA	TN	GNPS	TGW	GYPP	BMPP
Gene (G)	25	2684.4 **	408.8 **	220.2 **	283.9 **	17.4 **	796.1 **	244.6 **	78.5 **	400.3 **
Year (Y)	1	7.4	85.1 **	99.9 **	22,921.5 **	2456.4 **	7705.6 **	1237.0 **	302.6 **	11,775.3 **
Water (W)	1	4040.1 **	802.6 **	482.5 **	2719.1 **	168.6 **	2245.5 **	159.4 **	1235.8 **	7204.3 **
G×W	25	53 **	10.3	6.9	17.8	1	19.8	2.1 **	6 **	45.9 *
Y×W	1	25.1	3.6	0.1	110.5 **	30.8 **	0.8	0.8	9.7	16.1
G×Y	25	175.7 **	39.6 **	41.6 **	118.6 **	12.6 **	83.3 **	47 **	29.1 **	285.4 **
G×Y×W	25	26.4 *	4.7	4.8	27.2 *	1.2	21.5	1.6 **	7.8 **	42.2
Error	103	13.7	6.5	4.7	14.3	2.2	18.8	0.6	2.9	27.6
*H* ^2^		0.93	0.89	0.82	0.53	0.54	0.89	0.84	0.62	0.56

D.F., Degrees of freedom; *H*^2^, broad-sense heritability; PH, plant height; UIL, length of the first internodes under the spike; DSL, distance from spike to flag leaf ligule; FLA, area of flag; TN, tiller number; GNPS, number of grains spike^−1^; TGW, thousand-grain weight; GYPP, grain yield plant^−1^; BMPP, biomass yield plant^−1^; * and ** indicated significance at (*p* < 0.05) and (*p* < 0.01) levels, Duncan, respectively.

**Table 3:** Drought coefficient (DC) of agronomic traits of SHWs in two growth seasons.

Year	PH	UIL	DSL	FLA	TN	GNPS	TGW	GYPP	BMPP
2022–2023	0.93	0.89	0.85	0.75	0.78	0.86	0.96	0.58	0.63
2023–2024	0.92	0.91	0.84	0.73	0.76	0.89	0.95	0.7	0.76

**Table 4:** Photosynthetic parameters of flag leaves in three drought resistance groups (classified based on MFVD) at three key growth stages under WW and WS conditions.

WT	Growth Stage	Group	Pn (μmol CO_2_ m^−2^ s^−1^)	WUE (μmol mmol^−1^)	Ls	SPAD
WW	Heading	Group I	22.36 ± 3.47 Aa	4.23 ± 2.68 Aa	0.37 ± 0.12 Aa	50.09 ± 5.09 Aa
		Group II	21.67 ± 4.94 Aa	4.5 ± 2.17 Aa	0.43 ± 0.14 Aa	50.21 ± 4.80 Aa
		Group III	23.52 ± 4.01 Aa	4.3 ± 1.45 Aa	0.4 ± 0.1 Aa	52.61 ± 5.22 Aa
	Flowering	Group I	22.37 ± 3.74 Aa	4.79 ± 0.73 Aa	0.38 ± 0.06 Aab	55.01 ± 5.55 Aab
		Group II	21.99 ± 3.73 Aa	4.91 ± 1.22 Aa	0.42 ± 0.08 Aa	52.77 ± 5.76 Ab
		Group III	23.97 ± 4.21 Aa	4.87 ± 0.85 Aa	0.37 ± 0.06 Ab	57.69 ± 5.43 Aa
	Grain-filling	Group I	21.05 ± 5.5 Aa	5.26 ± 2.03 Aa	0.36 ± 0.13 Aa	54.06 ± 5.36 Aa
		Group II	20.9 ± 3.95 Aa	5.77 ± 2.54 Aa	0.41 ± 0.13 Aa	53.78 ± 5.11 Aa
		Group III	24.09 ± 4.19 Aa	5.86 ± 3.66 Aa	0.36 ± 0.09 Aa	56.27 ± 6.78 Aa
WS	Heading	Group I	13.38 ± 3.25 Bc	4.92 ± 1 Aa	0.56 ± 0.12 Aa	46.64 ± 4.4 Ab
		Group II	15.82 ± 2.98 Bb	5.21 ± 1.36 Aa	0.52 ± 0.12 Aab	47.99 ± 4.82 Aab
		Group III	19.15 ± 3.26 Aa	5.14 ± 1.94 Aa	0.46 ± 0.13 Ab	51.03 ± 5.34 Aa
	Flowering	Group I	14.18 ± 2.37 Bc	5.95 ± 1.15 Aa	0.49 ± 0.09 Aa	49.13 ± 3.51 Ab
		Group II	16.64 ± 2.64 ABb	5.58 ± 0.82 Aa	0.45 ± 0.07 Aab	50.6 ± 4.04 Aab
		Group III	19.08 ± 3.41 Aa	5.88 ± 0.91 Aa	0.43 ± 0.05 Ab	53.01 ± 4.57 Aa
	Grain-filling	Group I	13.73 ± 3.17 Bb	6.78 ± 3.65 Aa	0.58 ± 0.2 Aa	51.99 ± 4.94 Aab
		Group II	16.15 ± 2.94 Bb	7.16 ± 2.36 Aa	0.48 ± 0.16 Aa	51.45 ± 4.73 Ab
		Group III	19.76 ± 4.38 Aa	7.04 ± 2.08 Aa	0.46 ± 0.15 Aa	55.56 ± 6.54 Aa

Data were presented as mean ± SD (standard deviation). WT, water treatment; WW, well-watered; WS, water-stressed; Pn, Net photosynthetic rate; Ls, Stomatal limitation value; WUE, water utilization efficiency; SPAD, chlorophyll relative content. Different lowercase and uppercase letters represent different significance among the different water conditions at (*p* < 0.05) and (*p* < 0.01).

**Table 5:** The fluorescence parameter in the flag leaf at three main growth stages of the three different drought resistance groups of study materials based on MFVD under WW and WS treatment.

WT	Growth Stage	Group	Fv/Fm	Fv′/Fm′	ΦPSII	qP	qN	ETR
WW	Heading	Group I	0.84 ± 0.01 Aa	0.64 ± 0.06 Aa	0.21 ± 0.06 Aa	0.37 ± 0.24 Aa	0.61 ± 0.19 Aa	103.56 ± 29.04 Aa
		Group II	0.84 ± 0.02 Aa	0.64 ± 0.09 Aa	0.23 ± 0.06 Aa	0.39 ± 0.13 Aa	0.65 ± 0.13 Aa	117.33 ± 32.83 Aa
		Group III	0.84 ± 0.01 Aa	0.63 ± 0.08 Aa	0.26 ± 0.1 Aa	0.43 ± 0.19 Aa	0.68 ± 0.12 Aa	131.32 ± 50.47 Aa
	Flowering	Group I	0.83 ± 0.13 Aa	0.68 ± 0.38 Aa	0.23 ± 0.06 Aa	0.39 ± 0.13 Aa	0.64 ± 0.14 Aa	116.99 ± 30.86 Aa
		Group II	0.81 ± 0.08 Aa	0.68 ± 0.42 Aa	0.23 ± 0.05 Aa	0.43 ± 0.2 Aa	0.64 ± 0.19 Aa	117.72 ± 25.76 Aa
		Group III	0.83 ± 0.06 Aa	0.67 ± 0.44 Aa	0.24 ± 0.09 Aa	0.39 ± 0.15 Aa	0.61 ± 0.17 Aa	119.25 ± 43.22 Aa
	Grain-filling	Group I	0.82 ± 0.07 Aa	0.65 ± 0.16 Aa	0.24 ± 0.06 Aa	0.38 ± 0.12 Aa	0.65 ± 0.1 Aa	118.9 ± 28.86 Aa
		Group II	0.8 ± 0.13 Aa	0.65 ± 0.5 Aa	0.25 ± 0.09 Aa	0.41 ± 0.18 Aa	0.62 ± 0.14 Aa	115.45 ± 29.07 Aa
		Group III	0.83 ± 0.07 Aa	0.67 ± 0.51 Aa	0.23 ± 0.06 Aa	0.39 ± 0.13 Aa	0.69 ± 0.1 Aa	127.39 ± 48.16 Aa
WS	Heading	Group I	0.84 ± 0.01 Aa	0.61 ± 0.1 Aa	0.17 ± 0.08 Bb	0.31 ± 0.19 Ab	0.68 ± 0.12 Aa	87.55 ± 41.19 Bb
		Group II	0.84 ± 0.01 Aa	0.63 ± 0.09 Aa	0.2 ± 0.04 ABb	0.34 ± 0.13 Aab	0.64 ± 0.15 Aa	101.93 ± 22.46 ABb
		Group III	0.84 ± 0.01 Aa	0.62 ± 0.14 Aa	0.25 ± 0.07 Aa	0.54 ± 0.52 Aa	0.7 ± 0.14 Aa	127.1 ± 37.21 Aa
	Flowering	Group I	0.83 ± 0.01 Aa	0.68 ± 0.07 Aa	0.2 ± 0.08 Aa	0.36 ± 0.12 Aa	0.51 ± 0.18 Aa	99.67 ± 24.99 Aa
		Group II	0.83 ± 0.01 Aa	0.66 ± 0.08 Aa	0.2 ± 0.05 Aa	0.31 ± 0.12 Aa	0.53 ± 0.16 Aa	106.03 ± 32.77 Aa
		Group III	0.82 ± 0.02 Aa	0.66 ± 0.08 Aa	0.24 ± 0.07 Aa	0.35 ± 0.15 Aa	0.57 ± 0.16 Aa	120.01 ± 41.51 Aa
	Grain-filling	Group I	0.84 ± 0.01 Aa	0.67 ± 0.06 Aa	0.2 ± 0.05 Ab	0.3 ± 0.07 Bb	0.66 ± 0.07 Aa	99.55 ± 23.45 Bb
		Group II	0.84 ± 0.01 Aa	0.65 ± 0.07 Aa	0.2 ± 0.04 Ab	0.31 ± 0.09 ABb	0.64 ± 0.11 Aa	99.04 ± 18.59 Bb
		Group III	0.84 ± 0.01 Aa	0.65 ± 0.05 Aa	0.24 ± 0.05 Aa	0.38 ± 0.08 Aa	0.66 ± 0.08 Aa	120.71 ± 24.27 Aa

Data were presented as mean ± SD (standard deviation). WT, water treatment; WW, well-watered; WS, water-stressed; Fv′/Fm′, Excitation energy capture efficiency of PSII; ΦPSII, Actual photochemical quantum efficiency of PSII; qP, Photochemical quenching coefficient; qN, non-photochemical quenching coefficient; ETR, Rate of linear electron transport in PSII at given PAR. Different lowercase and uppercase letters represent different significance among the different water conditions at (*p* < 0.05) and (*p* < 0.01).

**Table 6:** Simple correlation coefficients (r) between MFVD with net photosynthetic rate, stomatal limitation in WW and WS treatments.

WT	Growth Stages	Item								
		Pn	Ls	Fv′/Fm′	ΦPSII	qP	qN	ETR	WUE	Fv/Fm
WW	heading	0.073	0.049	0.027	0.175	0.02	0.069	0.18	−0.013	0.005
	flowering	0.221	−0.076	0.169	0.02	−0.111	−0.156	0.032	0.009	−0.106
	grain-filling	0.185	−0.092	−0.103	0.035	0.111	0.052	0.034	−0.021	0.062
WS	heading	0.653 **	−0.292 *	0.032	0.279 *	0.127	−0.063	0.285 *	0.078	0.141
	flowering	0.639 **	−0.351 *	−0.197	−0.073	0.011	0.142	−0.089	−0.103	0.338 *
	grain-filling	0.631 **	−0.251	−0.116	0.283 *	0.277 *	−0.005	0.273 *	−0.154	0.242

MFVD, membership function value of drought resistance; Pn, net photosynthetic rate; Ls, stomatal limitation value; Fv′/Fm′, excitation energy capture efficiency of PSII; ΦPSII, actual photochemical quantum efficiency of PSII; qP, photochemical quenching coefficient; qN, non-photochemical quenching coefficient; ETR, rate of linear electron transport in PSII at given PAR; WUE, water utilization efficiency; Fv/Fm, maximum photochemical quantum efficiency of PSII; * and **, correlation is significant at the (*p* < 0.05) and (*p* < 0.01) levels, respectively.

**Table 7:** Simple correlation coefficients (r) between MFVD with the activities of the three antioxidant enzymes and chlorophyll content in the flag leaf in WW and WS treatments.

WT	Growth Stages	Item			
		SOD	CAT	POD	SPAD
WW	heading	0.399 **	0.406 **	0.450 **	0.220
	flowering	0.227	0.456 **	0.491 **	0182
	grain-filling	0.168	0.547 **	0.512 **	0.179
WS	heading	0.538 **	0.558 **	0.529 **	0.327 *
	flowering	0.586 **	0.602 **	0.564 **	0.351 *
	grain-filling	0.596 **	0.694 **	0.602 **	0.388 **

MFVD, membership function value of drought resistance; WT, water treatment; WW, well-watered; WS, water-stressed; the enzyme and SPAD value name with subscript indicates the activity of the enzyme and chlorophyll content at heading, flowering, and grain-filling stage; * and **, correlation is significance at the (*p* < 0.05) and (*p* < 0.01) level, respectively.

**Table 8:** Materials used in this study.

Code	Accession	Description	Chromosome Configuration	Chromosome Number
1	Chinese Spring ^BC^	Triticum aestivum	AABBDD	42
2	Xinong389 ^BC^	Triticum aestivum	AABBDD	42
3	Zhumai762 ^BC^	Triticum aestivum	AABBDD	42
4	Jinmai47 ^BC^	Triticum aestivum	AABBDD	42
5	Langdon/KU2039 ^SHW^	Synthetic hexaploid wheat	AABBDD	42
6	Langdon/KU2097 ^SHW^	Synthetic hexaploid wheat	AABBDD	42
7	Langdon/KU2159 ^SHW^	Synthetic hexaploid wheat	AABBDD	42
8	Langdon/KU2829 A ^SHW^	Synthetic hexaploid wheat	AABBDD	42
9	TACBOW0224 ^CA^	Chinese Spring-Elymus trachycaulus Disomic addition translocation chromosome T1SL-7SL	21″ + 1″T1SL-7SL	44
10	TACBOW0225 ^CA^	Chinese Spring-Elymus trachycaulus Disomic addition translocation chromosome T2HS-5HL	21″ + 1″T2HS-5HL	44
11	TACBOW0265 ^CA^	Chinese Spring-Elymus ciliaris Disomic addition 1Yc	21″ + 1″1Yc	44
12	TACBOW0299 ^CA^	Chinese Spring-Elymus ciliaris Disomic addition ?Sc	21″ + 1″?Sc	44
13	TACBOW0059 ^CA^	Chinese Spring-Haynaldia villosa Disomic 3V addition	21″ + 1″3V	44
14	TACBOW0213 ^CA^	Chinese Spring-Agropyron elongatum Disomic addition ditelosome 1ES	21″ + t″1ES	44
15	TACBOW0190 ^CA^	Chinese Spring-Aegilops caudata Disomic ?C addition	21″ + 1″?C	44
16	TACBOW0124 ^CA^	Chinese Spring-Leymus mollis Disomic addition ?G	21″ + 1″?G	44
17	TACBOW0018 ^CA^	Chinese Spring-Rye Disomic addition 1R	21″ + 1″1R	44
18	TACBOW0029 ^CA^	Chinese Spring-Rye Disomic addition 5R	21″ + 1″5R	44
19	TACBOW0066 ^HT^	Hexaploid Triticale	AABBRR	42
20	TACBOW0067 ^HT^	Hexaploid Triticale	AABBRR	42
21	Certa ^HT^	Hexaploid Triticale	AABBRR	42
22	WOH45 ^HT^	Hexaploid Triticale	AABBRR	42
23	TACBOW0065 ^OT^	Octoploid Triticale	AABBDDRR	56
24	TACBOW0167 ^OT^	Octoploid Triticale	AABBDDRR	56
25	TACBOW0172 ^OT^	Octoploid Triticale	AABBDDRR	56
26	TACBOW0182 ^OT^	Octoploid Triticale	AABBDDRR	56

^BC^, bread wheat cultivars; ^SHW^, Synthetic hexaploid wheat; ^CA^, Chinese spring addition lines; ^HT^, Hexaploid Triticale; ^OT^, Octoploid Triticale.

## Data Availability

All of the data generated or analyzed during this study are included in this published article.

## Abbreviations

The following abbreviations are used in this manuscript:

WT	Water treat
WW	Well-watered
WS	Water-stressed
Pn	Net photosynthesis rate
WUE	Water use efficiency
Ls	Stomatal limitation value
ΦPSII	Actual photochemical quantum efficiency of photosystem II
qP	Coefficient of photochemical quenching
NPQ	Non-photochemical quenching
ETR	Electron transfer rate
CAT	Catalase
SOD	Superoxide dismutase
POD	Peroxidase
MFVD	Membership function value of drought resistance
DC	Drought-resistant coefficient
DI	Drought resistance index
PH	Plant height
UIL	Length of the first internode under the spike
DSL	Distance from spike to flag leaf ligule
FLA	Flag leaf area
TN	Tiller number
GNPS	Number of grains per spike
TGW	Thousand kernel weight
GYPP	Grain yield per plant
BMPP	Biomass per plant
ROS	Reactive oxygen species
*H*^2^	Broad sense heritability
BC	Bread wheat cultivars
CA	Disomic alien chromosome addition lines
OT	Octoploid Triticale lines
SHW	Synthetic hexaploid wheat lines
HT	Hexaploid Triticale lines
